# Temperature-dependent Schottky barrier in high-performance organic solar cells

**DOI:** 10.1038/srep40134

**Published:** 2017-01-10

**Authors:** Hui Li, Dan He, Qing Zhou, Peng Mao, Jiamin Cao, Liming Ding, Jizheng Wang

**Affiliations:** 1 Beijing National Laboratory for Molecular Sciences, CAS Key Laboratory of Organic Solids, Institute of Chemistry, Chinese Academy of Sciences, Beijing 100190, P. R. China; 2 National Center for Nanoscience and Technology, Beijing 100190, P.R. China; 3 University of Chinese Academy of Sciences, Beijing 100049, China

## Abstract

Organic solar cells (OSCs) have attracted great attention in the past 30 years, and the power conversion efficiency (PCE) now reaches around 10%, largely owning to the rapid material developments. Meanwhile with the progress in the device performance, more and more interests are turning to understanding the fundamental physics inside the OSCs. In the conventional bulk-heterojunction architecture, only recently it is realized that the blend/cathode Schottky junction serves as the fundamental diode for the photovoltaic function. However, few researches have focused on such junctions, and their physical properties are far from being well-understood. In this paper based on PThBDTP:PC_71_BM blend, we fabricated OSCs with PCE exceeding 10%, and investigated temperature-dependent behaviors of the junction diodes by various characterization including current-voltage, capacitance-voltage and impedance measurements between 70 to 290 K. We found the Schottky barrier height exhibits large inhomogeneity, which can be described by two sets of Gaussian distributions.

The architecture development of organic solar cells (OSCs) has experienced three major steps, namely single-layer, bi-layer and bulk-heterojunction structures. Due to the intrinsic small dielectric constant of organic materials (usually about 2–4), the exciton binding energy is very large (0.1–0.5 eV)^1^,^2^, the thermal energy *k*T (which is only about 0.026 eV at room temperature, *k* is the Boltzmann constant and T is temperature) is too weak to separate such excitons into free electrons and holes. This is exactly the problem for the single layer device where a single organic semiconductor film is sandwiched between two electrodes with different workfunctions (cathode/organic layer/anode). Only very few photogenerated excitons can be dissociated by trap states in the film, which can capture either the electron or the hole and hence free the other in an exciton. The photovoltaic performance is seriously hampered by such limited free carriers, and the power conversion efficiency (PCE) stays very low at about 0.1%^2^,^3^. With the development of n and p type organic semiconductor materials, bilayer device structure was designed, where the interface of a p-type semiconductor layer and a n-type semiconductor layer serves as the border to dissociate excitons into free electrons and holes, and then the carriers are driven in their respective channels (p-material for holes and n-materials for electrons) by the build-in electric field to their respective electrodes. This greatly enhances the PCE to about 1%^4^. However, the exicton diffusion length is very short in organic materials, which is only 3–10 nm^5^,^6^,^7^,^8^. So only a very narrow region near the interface can contribute photo excitons to the p-n interface, and hence the performance is still seriously self-limited. Later polymer:fullerene nanoscale network was found and bulk-heterojunction device structure was designed: polymer:fullerene blend serves as the active layer, there are rich p/n interfaces now in the film and almost all photogenerated excitons can diffuse to their nearby polymer:fullerene interfaces and dissociate: holes are then transported in p-type materials (called donor) and electrons are transported in n-type materials (called acceptor)^9^. This greatly improves the PCE of OSCs. With the rapid material development, PCE now reaches around 10% for such blend systems^10^,^11^,^12^,^13^,^14^,^15^,^16^.

However, the fundamental unit in OSCs has long been conceptually misunderstood. Conventionally OSCs were treated as metal-insulator-metal (MIM) diodes, mainly due to the low conductivity of organic materials. In such a view, the work function difference of the cathode and the anode builds an almost homogeneous internal electric field across the device, which sweeps photo electrons and holes to their respective electrodes, leading to photovoltaic performance. In such simple MIM model, the open-circuit voltage should be independent on light intensity, which is in sharp contrary to the experimental observations^17^. Later by carefully investigating the dark current it was found that in the bilayer architecture, the fundamental unit is PN junction (p-organic semiconductor/n-organic semiconductor), the same as that for inorganics^18^,^19^. In the single layer device (single polymer film or polymer:fullerene blend film), the fundamental unit is semiconductor/metal Schottky junction^20^,^21^,^22^. For the most popular P3HT (poly(3-hexylthiophene)) system, in the single layer structure ITO (indium-tin-oxide)/PEDOT:PSS (poly(3,4-ethylenedioxythiophene):poly (styrenesulfonate))/P3HT/Al (aluminum), P3HT has a p doping concentration about 10^16^ cm^−3^, and it forms Schottky junction with Al cathode (it forms ohmic contact with ITO/PEDOT:PSS anode). The photovoltaic performance is from this Schottky diode, not previously assumed MIM diode. In a blend heterojunction system (ITO/PEDOT:PSS/P3HT:PCBM (phenyl-C61-butyric acid methyl ester)/Al), the blend can actually be treated as one material with modified bandgap and doping concentration, the fundamental unit is still the blend/cathode Schottky junction. Although now the blend/metal junction has been recognized as the real base for the photovoltaic performance, few researches have focused on systematically investigating the properties of such blend/metal junctions.

In this paper employing a high-performance donor PThBDTP^23^, we fabricated ITO/PEDOT:PSS/PThBDTP:PC_71_BM (phenyl-C71-butyric acid methyl ester)/Al based solar cells with three different surface treatments for the blend film: 1) directly deposit Al cathode on the film (the Al device). 2) spin a drop of methanol liquid on the film and then deposit Al cathode (the methanol/Al device). 3) spin a layer of PFN (poly[(9,9-bis(3′-(N,N-dimethylamino) propyl)−2,7-fluorene)-alt-2,7-(9,9–dioctylfl uorene)]) on the blend film, and then deposit Al cathode (the PFN/Al device). We carefully investigated the blend/cathode Schottky junctions by a variety of measurements including capacitance-voltage, current-voltage and impedance in a wide temperature range of 70 to 290 K. We found that the PFN device has less interface traps, less carrier recombination and longer carrier lifetime. We found for all the three types of devices, the blend/Al Schottky barrier displays quite large inhomogeneity and exhibits two sets of Gaussian distributions. Notably, a high PCE of 10.31% is achieved for the PFN device.

## Experimental Section

### Material

PThBDTP was homemade and its synthesis has been described previously^23^. PC_71_BM (ADS71BFA) was purchased from American Dye Source and PFN was purchased from 1-material, both of them were used as received. The blend PThBDTP:PC_71_BM (weight ratios 1:1.2 wt.%) was dissolved in 1,2-dichlorobenzene at solution concentration of 12 mg/ml, then 3 vol% DIO (1,8-diiodooctane) was added in the mixed solution. The PFN interlayer material was dissolved in methanol at various solution concentration from 0.2–1.0 mg/ml in the presence of a small amount of acetic acid (2 μl/ml).

### Device Fabrication

A 40 nm thick PEDOT:PSS (Baytron PVP Al 4083) layer was first spun on a cleaned indium-tin-oxide (ITO) glass substrate, and was dried at 140 °C for 10 min in air. The blend PThBDTP:PC_71_BM film at different rotation speeds (700–1100 rpm) was then spin-coated on PEDOT:PSS film in nitrogen glove box. Methanol solvent or PFN solution with various concentrations at 2500 rpm was subsequently spin-coated onto the PThBDTP:PC_71_BM film, followed by thermally evaporation of 100 nm Al electrode. The optimized PThBDTP: PC_71_BM film thickness is about 95 nm.

### J-V Characterization and EQE

*J-V* characteristics of the devices were measured with a computer-controlled Keithley 2400 source meter and Newport solar simulator (6279 NS) with 100 mW/cm^2^ illumination at room temperature in nitrogen glove box. EQE measurements were performed by using Oriel Instrument IQE-200 (Newport) in the atmosphere. Prior to the use of the light, the light intensity was calibrated using a mono-silicon detector produced by the National Renewable Energy Laboratory.

### Low-temperature Measurements

The sample was mounted onto a LN_2_-coolable sample stage inside a vacuum chamber and the closed-cycle cryostat Janis CCS-150 was allowed conducting experiments in the 70–290 K temperature range by providing high-pressure helium gas to the cold head with compressor.

### IS and C-V Measurements

The impedance spectroscopy (IS) and capacitance-voltage measurements were performed using a Zahner Zennium electrochemical workstation. IS measurements were measured in a frequency range of 1 Hz to 4 MHz with an oscillation amplitude of 20 mV. C-V measurements were recorded at a frequency of 1 kHz. The light source for IS measurements is Newport solar simulator (6279 NS) with 100 mW/cm^2^ illumination.

## Results and Discussion

### Device Parameters

**Figure 1:**
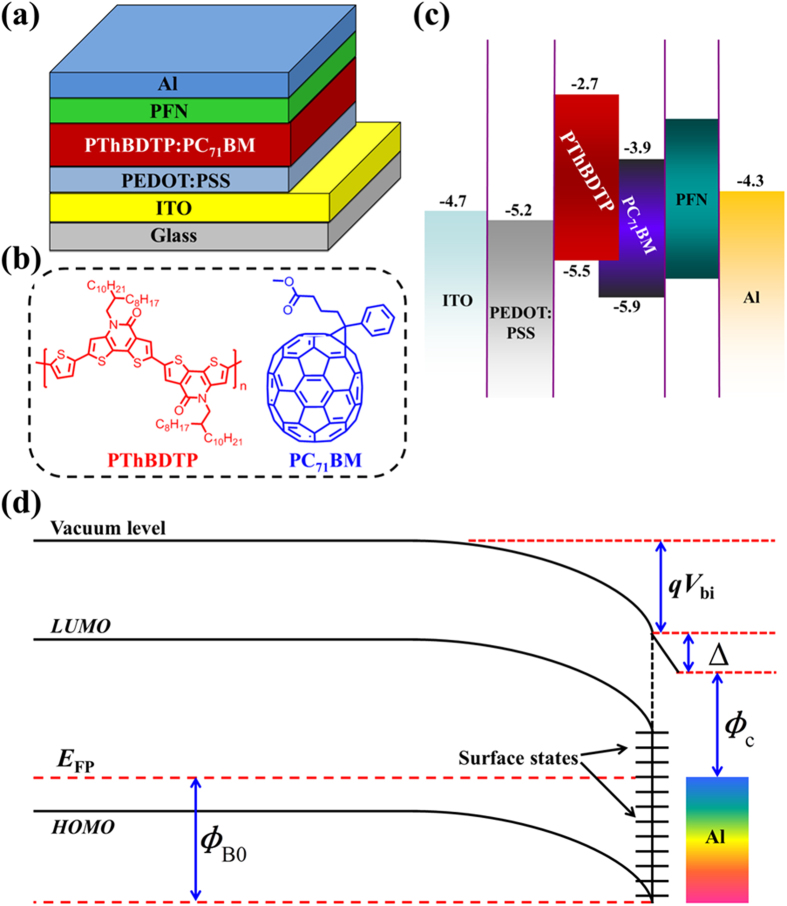
(**a**) Device structure. (**b**) Chemical structures of PThBDTP and PC_71_BM. (**c**) Energy level diagram. (**d**) Schematic diagram of energy levels and surface states.

**Figure 2:**
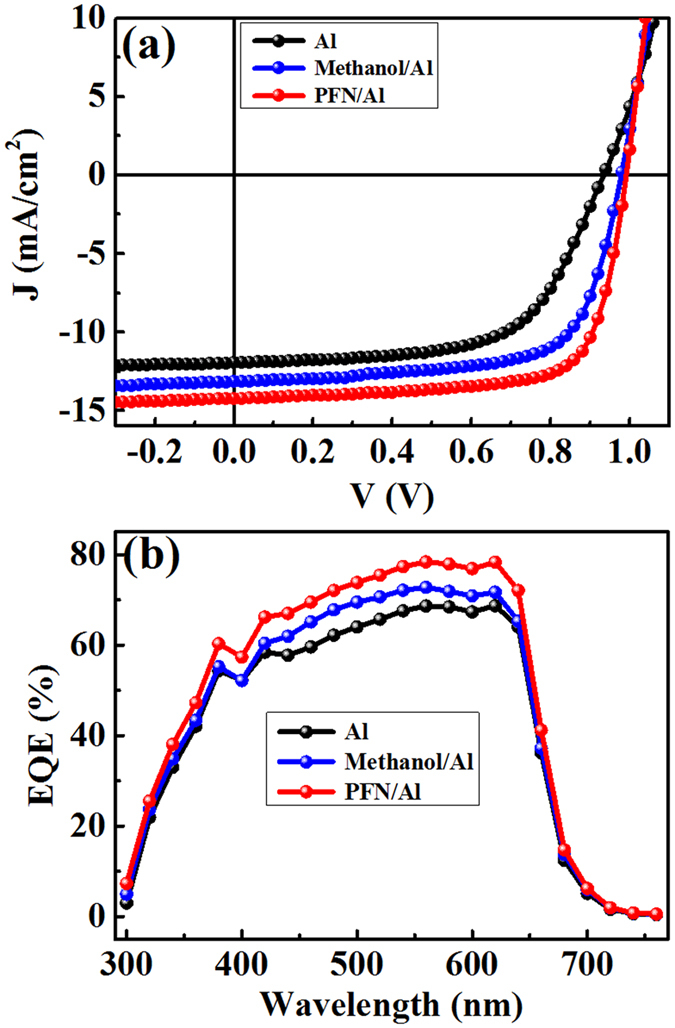
(**a**) *J* − *V* curves and (**b**) EQE spectra of the investigated devices.

Figure 1 presents the PFN device structure, material information and the band diagram. In our study, we firstly optimized the performances of all the devices by adjusting blend and PFN film thicknesses, which are shown in Figure S1 and Tables S1 and S2. Then we employed the best-performance devices for the low temperature study (shown in Figure S2 and Tables S3, S4 and S5). *J-V* (current density-voltage) and EQE (external quantum efficiency) curves of the selected best-performance devices at room temperature are shown in Fig. 2, and the corresponding device parameters are given in Table 1. The optimized PThBDTP:PC_71_BM device exhibits a PCE of 10.31%, with an open circuit voltage (*V*_oc_) of 0.990 V, a short-circuit current density (*J*_sc_) of 14.24 mA/cm^2^, and a fill factor (FF) of 73.1%. The average PCEs of 15 cells can reach 10.12%. The methanol/Al device shows a lower average PCE of 8.57%, and the Al device exhibits even lower average PCE of 6.65%: it is seen in Table 1 that *V*_oc_, *J*_sc_ and FF all drop, collectively leading to the PCE reduction.

**Figure 3:**
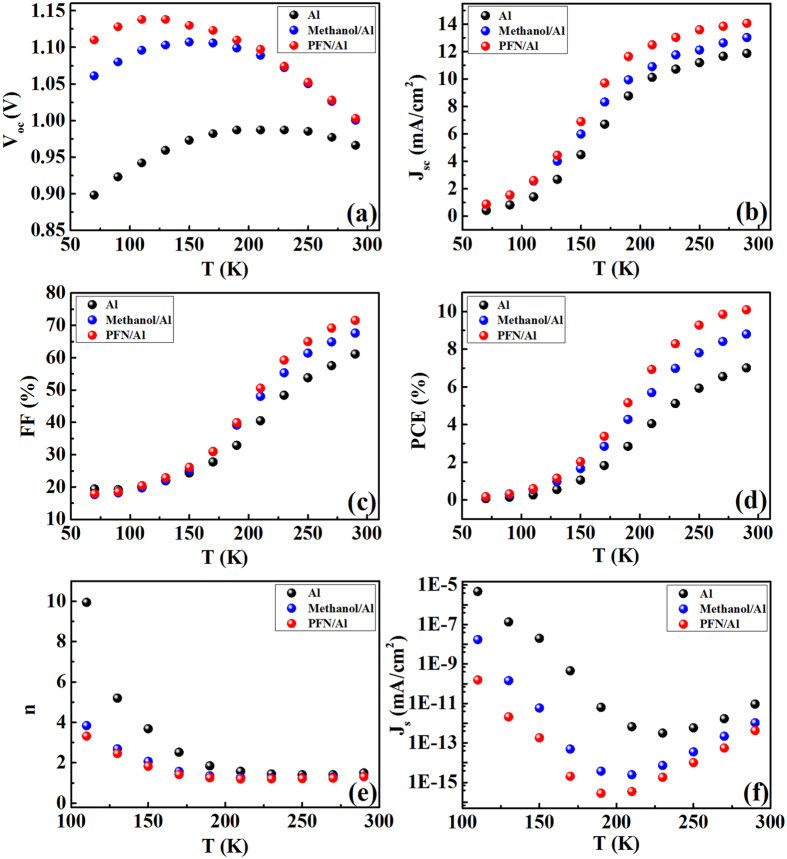
(**a**) *V*_oc_ (**b**) *J*_sc_ (**c**) FF (**d**) PCE (**e**) n and (**f**) *J*_s_ of the investigated devices at various temperatures.

The dark and light *J-V* curves at various temperatures are given in Figure S2, and the extracted parameters including *V*_oc_, *J*_sc_, FF, ideality factor n (extracted in dark *J-V* curves) and reverse saturation current density *J*_s_ (extracted in dark *J-V* curves) are listed in Tables S3, S4 and S5. The *V*_oc_-T, *J*_sc_-T, FF-T, PCE-T, n-T, *J*_s_-T curves are plotted in Fig. 3.

It is seen that the open-circuit voltage versus temperature shows an interesting trend: the measured open-circuit voltage increases with decreasing the temperature and then starts to saturate or even decrease. This can be explained by the trap states in the blend film. Trap states play a role of reducing the effective bandgap (the second term at the right side of Eq. 1)^24^,^25^:


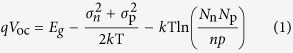


*q* is the elementary charge, *E*_g_ is the energy gap, *σ*_n_ (*σ*_p_) is the width of Gaussian density-of-states for the acceptor fullerene (donor polymer) (*σ* ≈ 100 meV at room temperature^24^,^25^), *N*_n_ (*N*_p_) is the effective conduction band (valence band) density-of-states, *n (p*) is the free electron (hole) concentration. For pure crystal without trap states, *V*_oc_ should increase linearly with decreasing temperature, caused by the third term (at the right side of Eq. 1), which represents carrier recombination (between the conduction band electrons and valence band holes) induced energy loss. For amorphous materials with rich traps the second term counts in, which is the tail states induced effective bandgap reduction. So temperature dependent *V*_oc_ becomes intrinsically dependent on the value of 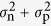, more disorders results in larger 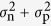, hence significantly reduces *V*_oc_ at low temperatures. The lower the T, the larger the second term. It is seen that *V*_oc_ of the Al device starts to drop at much higher temperature (170 K) in contrast to the other two type devices (130 K for the methanol/Al device and 90 K for the PFN/Al device), indicating more trap states inside the Al device, which should be caused by the Al atom that seeped into the blend film during the deposition of the Al cathode. For the PFN device, the Al atom could be somehow blocked by the PFN thin layer on top of the blend film.

The drop of *J*_sc_ and FF with decreasing temperature should be originated from the series resistance^26^, which is inversely proportional to carrier mobility: as temperature decreases, mobility in organic materials drops significantly, hence dramatically enhances series resistance, cutting both *J*_sc_ and FF. The reduced PCE with temperature is a collective result of the three parameters.

The ideality factor n presents very large values at low temperatures (much larger than 2), and *J*_s_ displays an interesting trend with decreasing the temperature: first decreases and then increases for all the three devices. As we know for an ideal Schottky junction, the barrier height usually should increase with decreasing the temperature, and the build-in voltage in the semiconductor side should also increase with decreasing the temperature^26^. And at low temperature, high-energy carriers that can surpass the barrier become less and less, so *J*_s_ should decrease with decreasing the temperature. The large n values and the abnormal *J*_s_ behavior with decreasing the temperature indicate that the Schottky junction is not ideal. We need to explore how the Schottky barrier is dependent on the temperature.

### Temperature-dependent Schottky Barrier and Gaussian Distribution

As we know, based on the Thermal Emission Theroy (TE), the saturation current density *J*_s_ can be expressed as the following equation^27^,^28^,^29^:


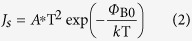


A* is the effective Richardson constant, and *Φ*_B0_ is the zero-bias barrier height (in eV).

It can be rewritten as


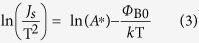


As shown in Figure S3, ln(*J*_s_/T^2^) is linearly dependent on 1/nT but not on 1/T. This is usually induced by temperature dependent barrier height and ideality factor, which can be explained by the lateral inhomogeneity of the Metal-Semiconductor Schottky barrier heights^30^,^31^. There are a number of factors that can produce barrier inhomogeneity such as non-uniformity of the interfacial charges, grain boundaries in the whole cathode contact area^30^,^32^. It is seen that the dependence of ln(*J*_s_/T^2^) on 1/nT displays two straight lines (110–150 K and 170–290 K) for all three kinds of devices, this indicates that there exists two sets of Gaussian distribution in the contact area.

The Gaussian distribution of barrier height can be represented by^33^,^34^,^35^:


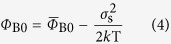


Where 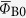 is the mean barrier height (in eV), *σ*_s_ (in eV) is the zero bias standard deviation of the Schottky barrier height distribution, and the larger the *σ*_s_, the more inhomogeneous the barrier is.

**Figure 4:**
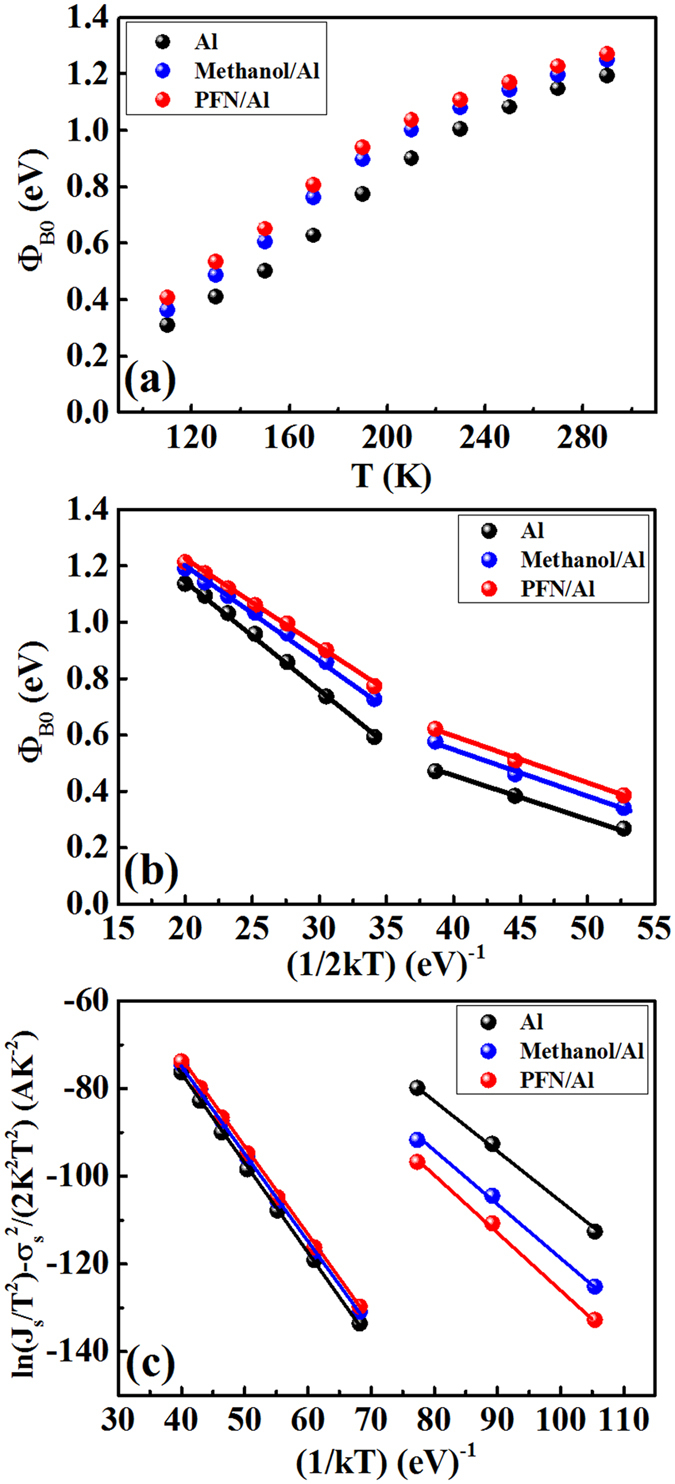
(**a**) Barrier height versus T of the investigated devices. (**b**) Barrier height versus 1/(2*k*T) and (**c**) ln(*J*_s_/T^2^) − (*σ*_s_^2^/2*k*^2^T^2^) versus 1/*k*T according to two Gaussian distributions of the barrier height.

The experimental values of *Φ*_B0_ calculated based on Eq. 2 are shown in Fig. 4a (assuming A* = 120 A/cm^2^K^2^). It is seen that with increasing the temperature, the barrier is increased. Such phenomenon should be induced by the Gaussian distribution of the barrier height. At low temperatures, carriers are only able to surmount lower barriers, so current transport is dominated by current flowing through the areas with lower barriers, which is concluded by a lower extracted barrier from the dark current. As the temperature increases, more and more carriers have sufficient energy to overcome higher barrier and as a result, the extracted barrier height increases with the temperature. From Eq. 4, 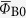 and *σ*_s_ can be obtained by fitting the *Φ*_B0_ − 1/2*k*T curve (Fig. 4b). The intercept is 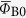 and the slope is *σ*_s_^2^. The extracted 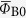 and *σ*_s_ are given in Table 2 for each devices. It is seen that for all the three devices 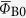 versus 1/2*k*T plot (Fig. 4c) have two linear regions, which correspond to two Gaussian distributions of barrier heights at two different temperature ranges: 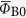 = 1.97–1.99 eV and *σ*_s_ = 0.18–0.21 eV in the high temperature range 170–290 K; and 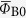 = 1.10–1.30 eV and *σ*_s_ = 0.12–0.13 eV in the low temperature range 110–150 K. It is seen that value of *σ*_s_ = 0.18–0.21 eV at high temperatures and *σ*_s_ = 0.12–0.13 eV at low temperatures are all quite large, indicating the large interface inhomogeneity of the blend/Al contacts. And at high temperatures the devices all exhibit much larger 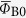 and *σ*_s_ than that at low temperatures, which should be originated by the enhanced thermal oscillation of the interface atoms and molecules. Furthermore, combining Eqs 3 and 4, Eq. 5 can be obtained:


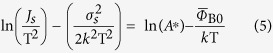


ln(*J*_s_/T^2^) − (σ_s_^2^/2*k*^2^T^2^) versus 1/*k*T (Fig. 4c) give a straight-line with slope represents the zero-bias mean 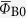 (Table S6): 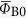 = 1.98–2.00 eV (in the range of 170–290 K) and 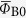 = 1.10–1.30 eV (in the range of 110–150 K). These values are very close to the 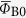 obtained from the 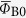 versus 1/2*k*T plot in Fig. 4b. And the extracted A* is about 120 A/cm^2^K^2^ (Table S6).

### Interface Charge and C-V Measurements

In order to gain information about the interface induced potential loss, we need to measure the build-in potential in the semiconductor side^20^,^36^:


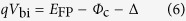


Here *V*_bi_ is built-in potential, *Φ*_c_ is cathode work function, Δ is the interface potential drop, and *E*_Fp_ is the hole Fermi level of the donor material. And then interface charge trapped by the interface states can be calculated based on the following equation^20^,^37^:


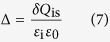


where *ε*_i_ is the dielectric constant of the interface layer (*ε*_i_ ∼ 3 for an organic layer), *ε*_0_ is the permittivity of the free space, and *δ* is the interface layer thickness (*δ* ∼ 5 Å).

*V*_bi_ can be extracted from the *C*-*V* measurements. *E*_Fp_ can be approximated by^20^


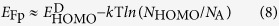


which assumes Boltzmann statistics for the hole occupancy of the HOMO levels. The density of states at the HOMO level is taken as *N*_HOMO_ ∼ 10^20^ cm^3^, and the background hole density caused by the doping can also be extracted from the *C-V* measurement (*N*_A_ ∼ 10^16^ cm^−3^).

**Figure 5:**
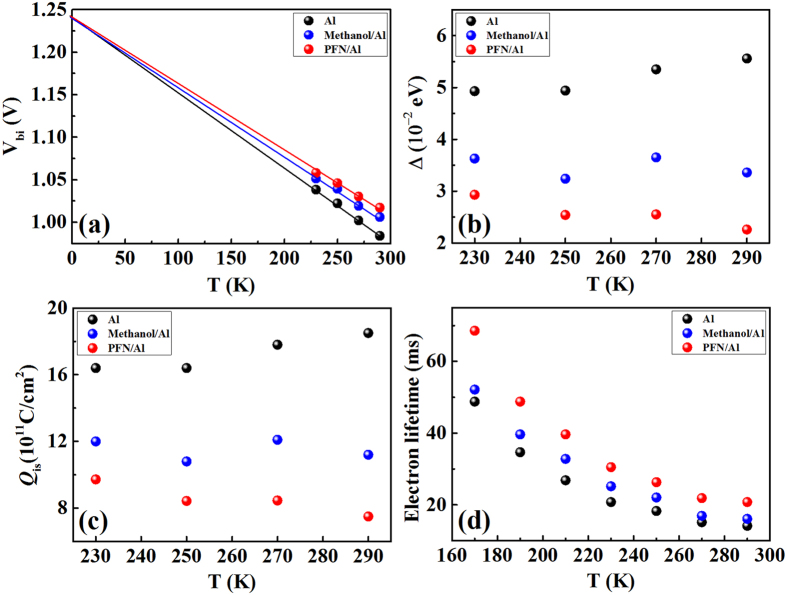
(**a**) Build-in potential (**b**) Interface potential drop (**c**) Interface charge and (**d**) electron lifetime of the investigated devices at various temperatures under the open circuit state in dark.

Figure S4 presents the Capacitance-Voltage (*C*^*−2*^-*V*) plots of the devices in the temperature range of 230 to 290 K (The capacitance cannot respond promptly with the AC signals at lower temperatures, which could be induced by the limited carrier mobility at low temperatures). From the plot, the built-in potential *V*_bi_ and doping concentration *N*_A_ can be extracted based on the Mott-Schottky equation^38^,^39^:


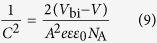


where *V* is the applied voltage, *A* is the device area. The extracted *V*_bi_, *N*_A_ and the calculated Δ and *Q*_is_ for the three devices are provided in Tables 3 and 4 and *V*_bi_, Δ, and *Q*_is_ versus temperature T are plotted in Fig. 5. It is seen that the PFN/Al device has the largest build-in potential and the least potential loss Δ and hence the least interface trap induced carrier accumulation. This is consistent with its superior performance over the Al device and the methanol/Al device. It is also seen that for all the three devices, the build-in potential *V*_bi_ increases linearly with decreasing the temperature, and the three *V*_bi_-T lines crosses at 0 K at a value of 1.24 eV, which represents the largest open-circuit voltage that can be achieved for the PThBDTP:PC_71_BM blend systems.

### Impedance Spectroscopy Measurement

Finally, we use Impedance spectroscopy to gain information about carrier lifetime and carrier recombination in the devices with a diffusion-recombination impedance model^38^,^40^ (open-circuit (the DC current is zero) and short-circuit (the bias voltage is zero) conditions under different temperatures and bias voltages in dark and in light.). The cole-cole plots of Z-Z’ are provided in Figures S5 and S6. The extracted lifetimes and recombination resistances with a diffusion-recombination impedance model^38^,^40^ are given in Figures S7 and S8 and Tables S7, S8, S9 and S10 (several other alternative techniques such as noise spectroscopy^41^,^42^ and open-circuit voltage decay^43^,^44^ can also be used to study the lifetimes and recombination resistances). Figure 5d presents the lifetime versus temperature in dark at open-circuit condition. It is seen that the lifetime increases with decreasing the temperature, this is induced by the reduced carrier number with decreasing the temperature. It is also seen that under various temperatures, the PFN/Al device exhibits the longest carrier lifetime. This is consistent with the conclusion that the PFN treatment offers the best interface between the blend and the Al cathode, which results in the best device performance.

In conclusion, ITO/PEDOT:PSS/PThBDTP:PC_71_BM/PFN/Al single junction organic solar cells with power conversion efficiency exceeding 10% were fabricated. Employing a variety of physical measurements, temperature dependent behaviors of the PFN device, the Al device and the methanol/Al device were systematically investigated. The results indicate that for all the three types of devices, the blend/Al Schottky barrier exhibits large inhomogeneity, which can be expressed by two sets of Gaussian distributions with large zero bias standard deviations. We compared performances of the three devices in details, and conclude that the role of PFN in enhancing the device efficiency is mainly reducing the blend surface states and blocking Al atoms to infiltrate into the blend film during the cathode deposition. It is worth noting that the Schottky barrier inhomogeneity was also observed in P3HT:PCBM, P3HT:ICBA (indene-C60 bisadduct), PTB7 (thieno[3,4-b]-thiophene/benzodithiophene): PCBM binary and P3HT:PCBM:ICBA ternary blend OSCs^45^.

## Additional Information

**How to cite this article:** Li, H. *et al*. Temperature-dependent Schottky barrier in high-performance organic solar cells. *Sci. Rep.* **7**, 40134; doi: 10.1038/srep40134 (2017).

**Publisher's note:** Springer Nature remains neutral with regard to jurisdictional claims in published maps and institutional affiliations.

## Supplementary Material

Supplementary Information

## Figures and Tables

**Table 1:** Parameters of the optimized devices.

Cathode	*V*_oc_ (V)	*J*_sc_ (mA/cm^2^)	FF (%)	PCE (%)	PCE_ave_ (%)
Al	0.933	11.96	61.6	6.87	6.65
Methanol/Al	0.978	13.17	68.2	8.78	8.57
PFN/Al	0.990	14.24	73.1	10.31	10.12

**Table 2:** Gaussian distributions at high (170–290 K) and low (110–150 K) temperatures.

Cathode	Al		Methanol/Al		PFN/Al	
T (K)	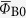 (eV)	*δ*_s_(eV)	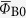 (eV)	*δ*_*s*_(eV)	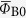 (eV)	*δ*_s_(eV)
High	1.985	0.202	1.979	0.188	1.977	0.182
Low	1.103	0.125	1.232	0.128	1.298	0.129

**Table 3:** Build-in potential and doping concentration.

Cathode	Al		Methanol/Al		PFN/Al	
T (K)	*V*_bi_ (V)	*N*_A_ (cm^−3^)	*V*_bi_ (V)	*N*_A_ (cm^−3^)	*V*_bi_ (V)	*N*_A_ (cm^−3^)
290	0.984	1.76 × 10^16^	1.006	5.55 × 10^16^	1.017	8.84 × 10^16^
270	1.002	1.64 × 10^16^	1.019	4.96 × 10^16^	1.030	7.27 × 10^16^
250	1.022	1.59 × 10^16^	1.039	4.42 × 10^16^	1.046	6.33 × 10^16^
230	1.038	1.36 × 10^16^	1.051	4.09 × 10^16^	1.058	5.62 × 10^16^

**Table 4:** Interface potential drop and interface charge.

Cathode	Al		Methanol/Al		PFN/Al	
T (K)	Δ (10^−2^ eV)	Q_is_ (10^12^ C/cm^2^)	Δ (10^−2^ eV)	Q_is_ (10^12^ C/cm^2^)	Δ (10^−2^ eV)	Q_is_ (10^11^ C/cm^2^)
290	5.56	1.85	3.36	1.12	2.26	7.50
270	5.35	1.78	3.65	1.21	2.55	8.46
250	4.94	1.64	3.24	1.08	2.54	8.43
230	4.93	1.64	3.63	1.20	2.93	9.72
Average	5.20	1.73	3.47	1.15	2.57	8.53
