# Supplementary material for: Temperature-dependent Schottky barrier in high-performance organic solar cells
Source: Sci Rep. 2017 Jan 10;7:40134. doi: 10.1038/srep40134 (PMC5223179; doi:10.1038/srep40134)
Supplement: Supplementary Information [file srep40134-s1.pdf]

Supporting Information

Temperature-dependent Schottky barrier in high-performance organic solar cells

Hui Li[1], Dan He[2], Qing Zhou[1], Peng Mao[1], Jiamin Cao[2], Liming Ding[2,3] & Jizheng Wang[1,3]

[1]Beijing National Laboratory for Molecular Sciences, CAS Key Laboratory of Organic Solids, Institute of Chemistry, Chinese Academy of Sciences, Beijing 100190, P. R. China
E-mail: jizheng@iccas.ac.cn

[2]National Center for Nanoscience and Technology, Beijing 100190, P.R. China,
Email: Ding@nanoctr.cn

[3]University of Chinese Academy of Sciences, Beijing 100049, China.
E-mail: jizheng@iccas.ac.cn, Ding@nanoctr.cn

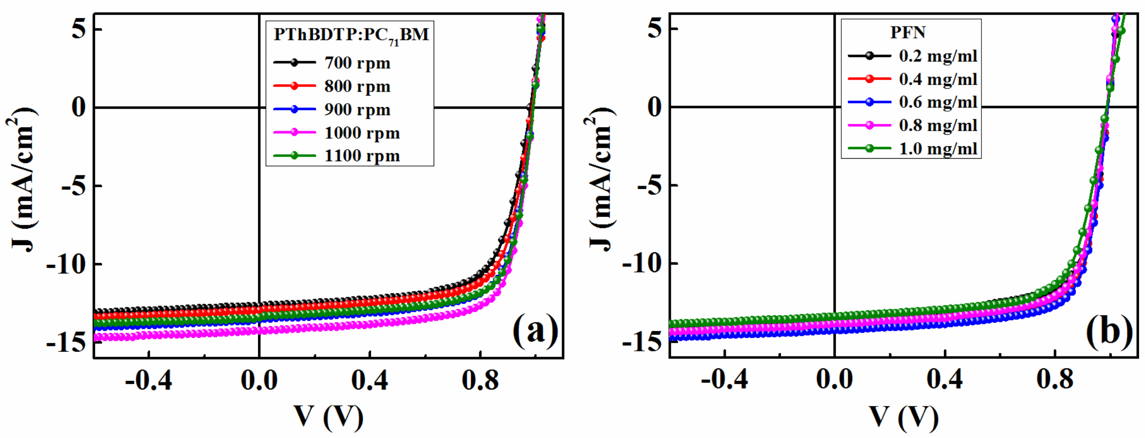

Figure S1 *J-V* curves of PThBDTP:$PC_{71}BM$ devices with (a) various active layer thicknesses (by adjusting rotation speed) at the fixed PFN buffer layer thickness (0.6 mg/ml) and (b) various PFN layer thicknesses (by adjusting solution concentration) at the fixed active layer thickness (1000 rpm)

Table S1 Parameters of the PThBDTP:$PC_{71}BM$ devices with various active layer thicknesses (by adjusting rotation speed) at the fixed PFN buffer layer thickness (0.6 mg/ml).

PThBDTP:$PC_{71}BM$	V_{oc} (V)	J_{sc} (mA/cm^2)	FF (%)	PCE (%)
700 rpm	0.980	12.66	69.3	8.60
800 rpm	0.986	12.95	70.7	9.03
900 rpm	0.990	13.52	71.8	9.61
1000 rpm	0.990	14.24	73.1	10.31
1100 rpm	0.991	13.38	72.8	9.65

Table S2 Parameters of the PThBDTP:$PC_{71}BM$ devices with various PFN layer thicknesses (by adjusting solution concentration) at the fixed active layer thickness (1000 rpm).

PFN	V_{oc} (V)	J_{sc} (mA/cm^2)	FF (%)	PCE (%)
0.2 mg/ml	0.990	13.55	69.9	9.38
0.4 mg/ml	0.989	13.92	72.4	9.97
0.6 mg/ml	0.990	14.24	73.1	10.31
0.8 mg/ml	0.987	13.80	71.4	9.73
1.0 mg/ml	0.988	13.37	68.9	9.10

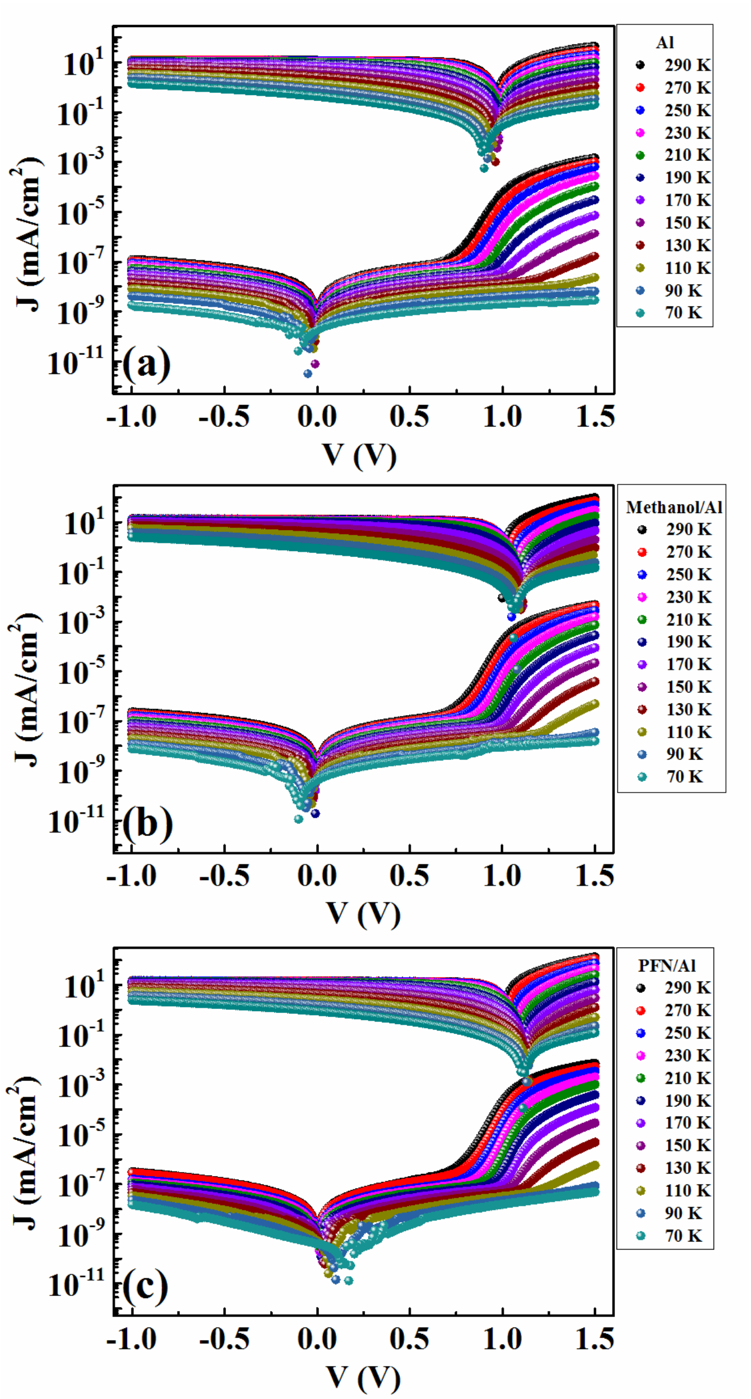

Figure S2 *J-V* curves of PThBDTP:$PC_{71}BM$ based devices under illumination and in dark in the temperature range of 70-290 K: (a) the Al device. (b) the methanol/Al device and (c) the PFN/Al device.

Table S3 Parameters of the PThBDTP:$PC_{71}BM$ based device without cathode buffer layer (the Al device).

Al	V_{oc} (V)	J_{sc} (mA/cm^2)	FF (%)	PCE (%)	n	J_s (mA/cm^2)
290 K	0.966	11.87	61.1	7.01	1.50	9.36×10^{-12}
270 K	0.977	11.66	57.5	6.55	1.41	1.71×10^{-12}
250 K	0.985	11.19	53.8	5.93	1.41	5.88×10^{-13}
230 K	0.987	10.72	48.4	5.12	1.45	3.19×10^{-13}
210 K	0.987	10.12	40.5	4.05	1.58	6.70×10^{-13}
190 K	0.987	8.77	32.9	2.85	1.85	6.38×10^{-12}
170 K	0.982	6.71	27.8	1.83	2.52	4.54×10^{-10}
150 K	0.973	4.49	24.4	1.07	3.69	2.00×10^{-8}
130 K	0.959	2.69	21.9	0.56	5.20	1.35×10^{-7}
110 K	0.942	1.42	20.2	0.27	9.94	4.74×10^{-6}
90 K	0.923	0.83	19.2	0.15	-	-
70	0.898	0.43	19.4	0.075	-	-

Table S4 Parameters of the PThBDTP:$PC_{71}BM$ based device with methanol treatment (the methanol/Al device).

Methanol/Al	V_{oc} (V)	J_{sc} (mA/cm^2)	FF (%)	PCE (%)	n	J_s (mA/cm^2)
290 K	1.000	13.02	67.6	8.80	1.32	1.06×10^{-12}
270 K	1.026	12.63	64.9	8.41	1.29	2.19×10^{-13}
250 K	1.050	12.11	61.4	7.81	1.26	3.57×10^{-14}
230 K	1.072	11.76	55.3	6.97	1.25	7.27×10^{-15}
210 K	1.089	10.90	48.1	5.71	1.27	2.46×10^{-15}
190 K	1.099	9.94	39.2	4.28	1.35	3.76×10^{-15}
170 K	1.106	8.32	30.9	2.84	1.57	4.92×10^{-14}
150 K	1.107	5.99	25.1	1.66	2.07	5.93×10^{-12}
130 K	1.103	4.02	22.2	0.98	2.69	1.44×10^{-10}
110 K	1.096	2.57	19.8	0.56	3.84	1.74×10^{-8}
90 K	1.080	1.54	18.2	0.30	-	-
70 K	1.061	0.87	17.7	0.16	-	-

Table S5 Parameters of the PThBDTP:$PC_{71}BM$ based device with PFN cathode buffer (the PFN/Al device).

PFN/Al	V_{oc} (V)	J_{sc} (mA/cm^2)	FF (%)	PCE (%)	n	J_s (mA/cm^2)
290 K	1.003	14.06	71.6	10.10	1.31	4.28×10^{-13}
270 K	1.028	13.84	69.2	9.85	1.24	5.57×10^{-14}
250 K	1.052	13.58	65.0	9.29	1.22	1.02×10^{-14}
230 K	1.074	13.03	59.3	8.30	1.21	1.84×10^{-15}
210 K	1.097	12.49	50.6	6.93	1.20	3.48×10^{-16}
190 K	1.110	11.64	39.9	5.16	1.26	2.84×10^{-16}
170 K	1.123	9.71	31.0	3.38	1.42	2.07×10^{-15}
150 K	1.130	6.90	26.1	2.04	1.83	1.84×10^{-13}
130 K	1.138	4.45	22.9	1.16	2.46	2.12×10^{-12}
110 K	1.138	2.61	20.5	0.61	3.32	1.57×10^{-10}
90 K	1.128	1.56	18.6	0.33	-	-
70 K	1.110	0.89	17.9	0.18	-	-

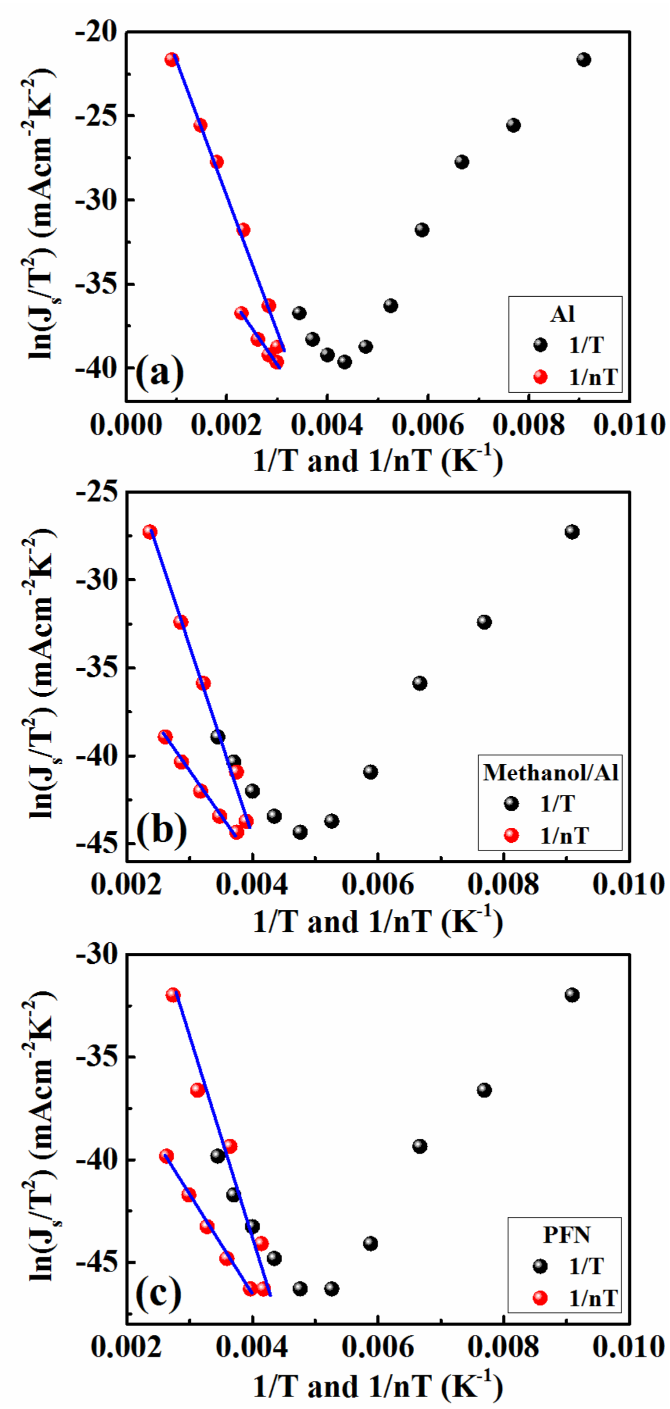

Figure S3 The plots of ln(J_s/T^2) with 1/T and 1/nT for the three kinds of devices: (a) the Al device. (b) the methanol/Al device and (c) the PFN/Al device.

Table S6 The $\bar{\Phi}_{B0}$ and A^* obtained from the equation: $\ln(\frac{J_s}{T^2}) - (\frac{\sigma_s^2}{2k^2T^2}) = \ln(A^*) - \frac{\bar{\Phi}_{B0}}{kT}$.

Cathode	Al		Methanol/Al		PFN/Al	
T (K)	$\bar{\Phi}_{B0}$ (eV)	A^* (A/cm^{2}K^2)	$\bar{\Phi}_{B0}$ (eV)	A^* (A/cm^{2}K^2)	$\bar{\Phi}_{B0}$ (eV)	A^* (A/cm^{2}K^2)
High	1.994	131	1.987	112	1.980	117
Low	1.101	136	1.225	108	1.299	124

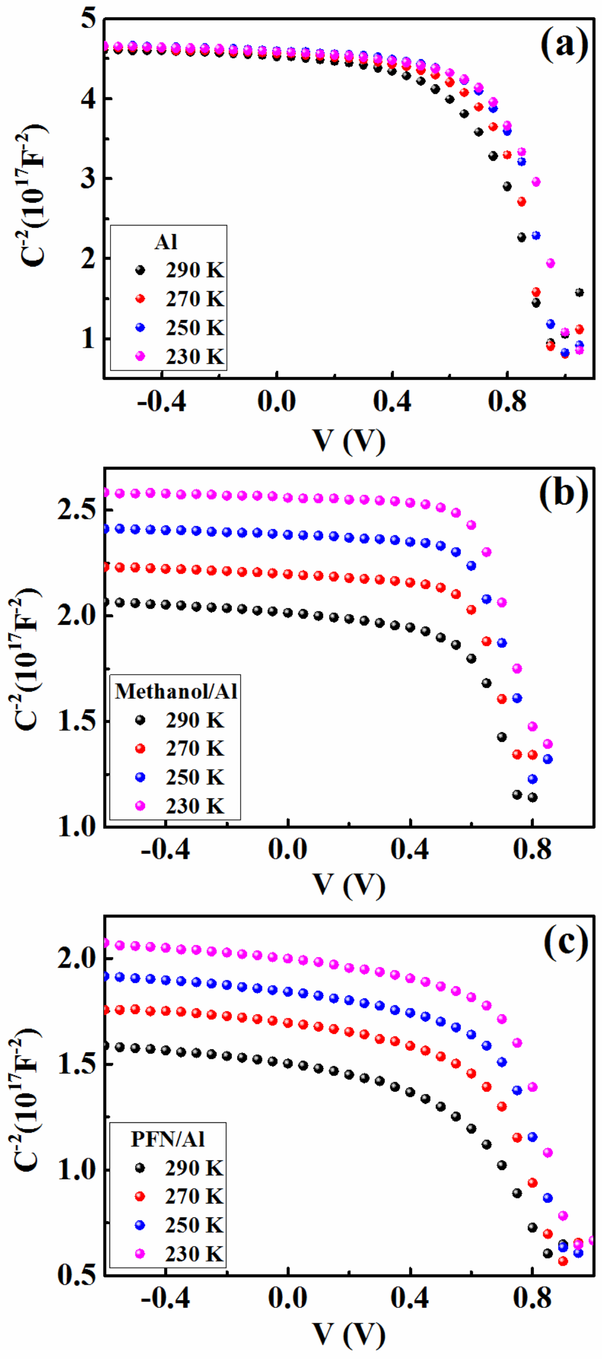

Figure S4 Capacitance-voltage analysis for the three kinds of devices: (a) the Al device. (b) the methanol/Al device and (c) the PFN/Al device.

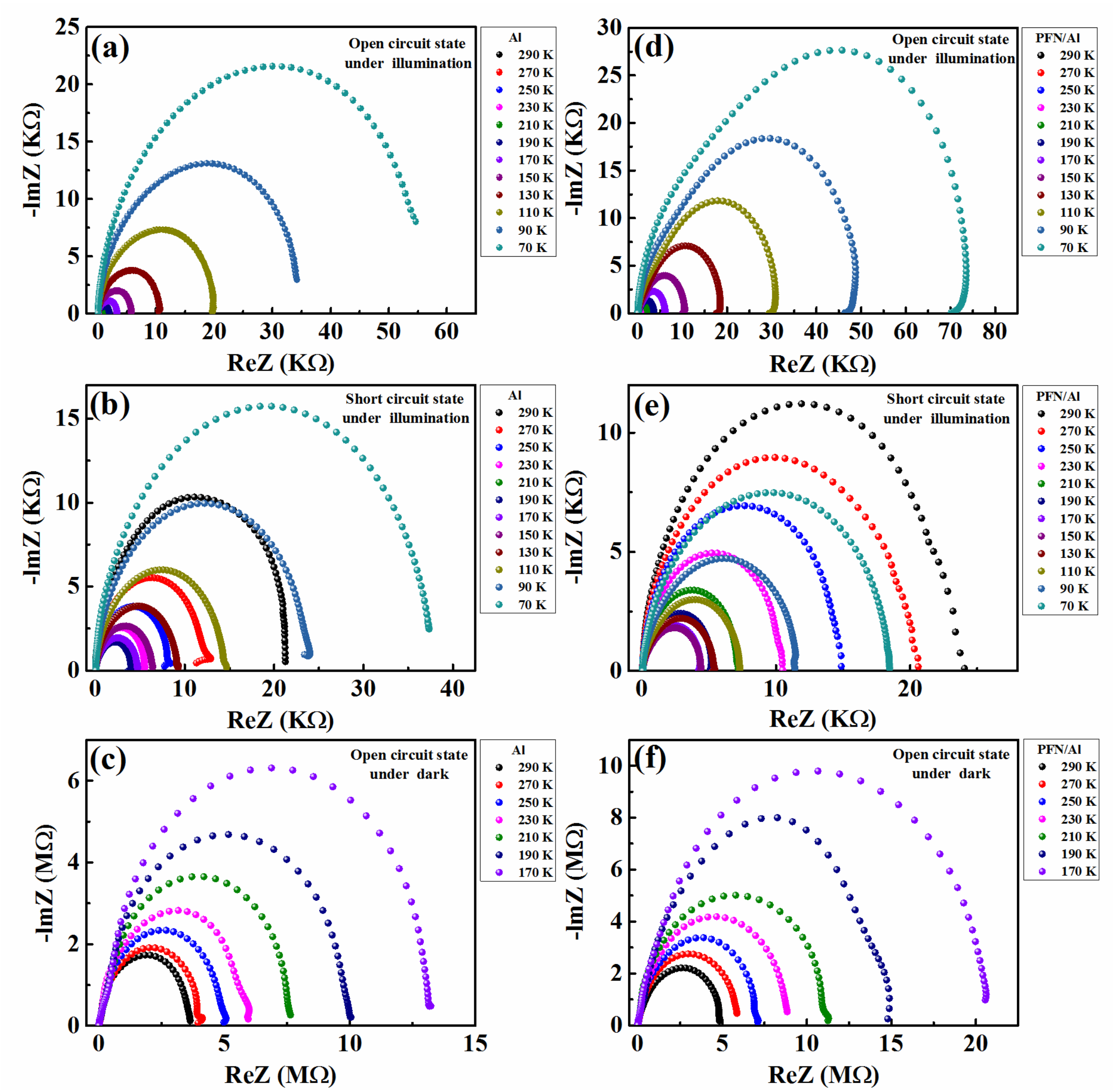

Figure S5 Impedance spectra measured at various temperatures for the Al and PFN/Al devices:(a) and (d) at open circuit state under illumination. (b) and (e) at short circuit state under illumination. (c) and (f) at open circuit state in dark.

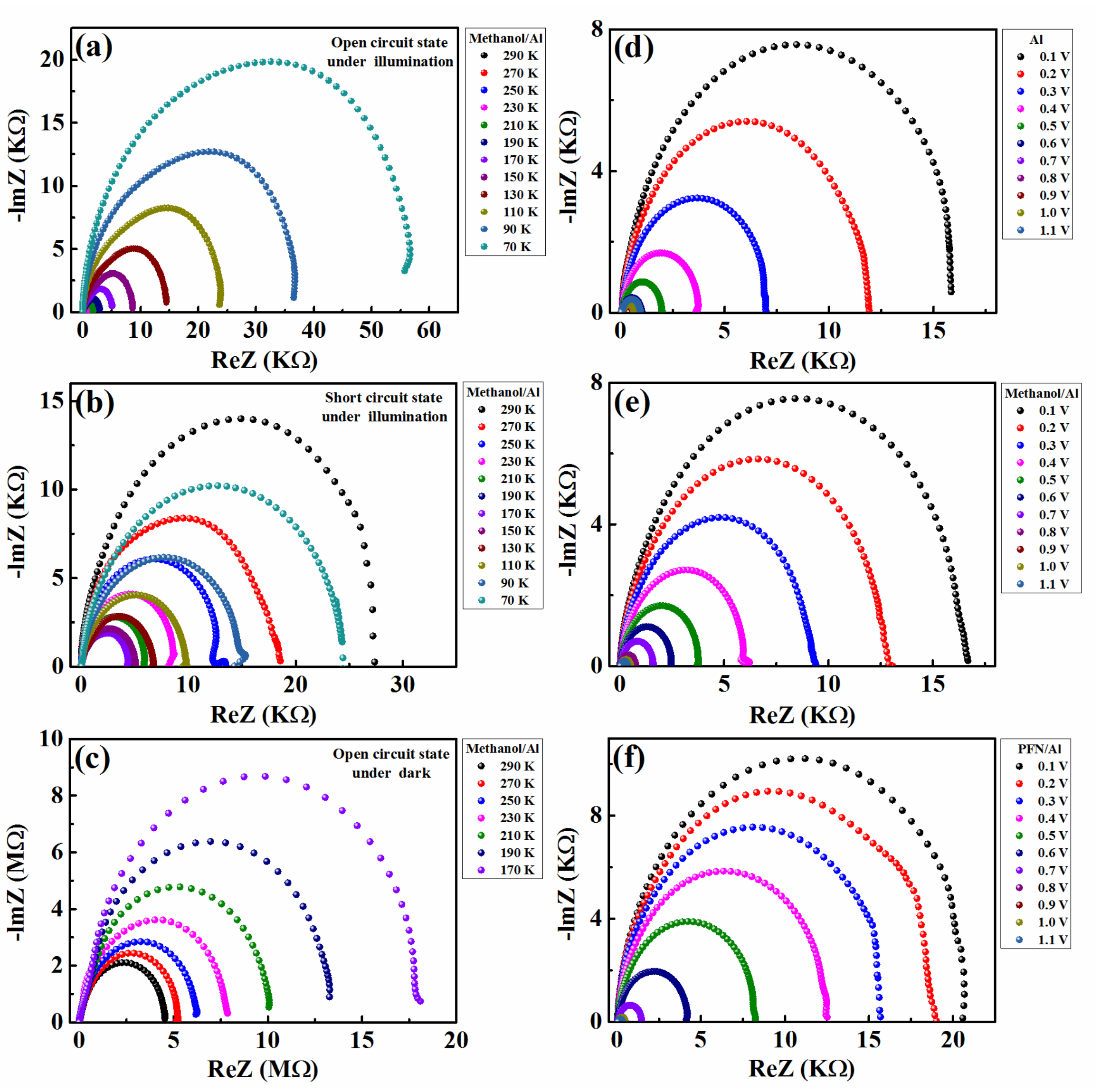

Figure S6 Impedance spectra measured at various temperatures for the methanol/Al device: (a) at open circuit state under illumination. (b) at short circuit state under illumination. (c) at open circuit state under dark. And Impedance spectra measured at various bias voltages under illumination at fixed 290 K temperature for the three kinds of devices: (d) the Al device. (e) the methanol/Al device. (f) the PFN/Al device.

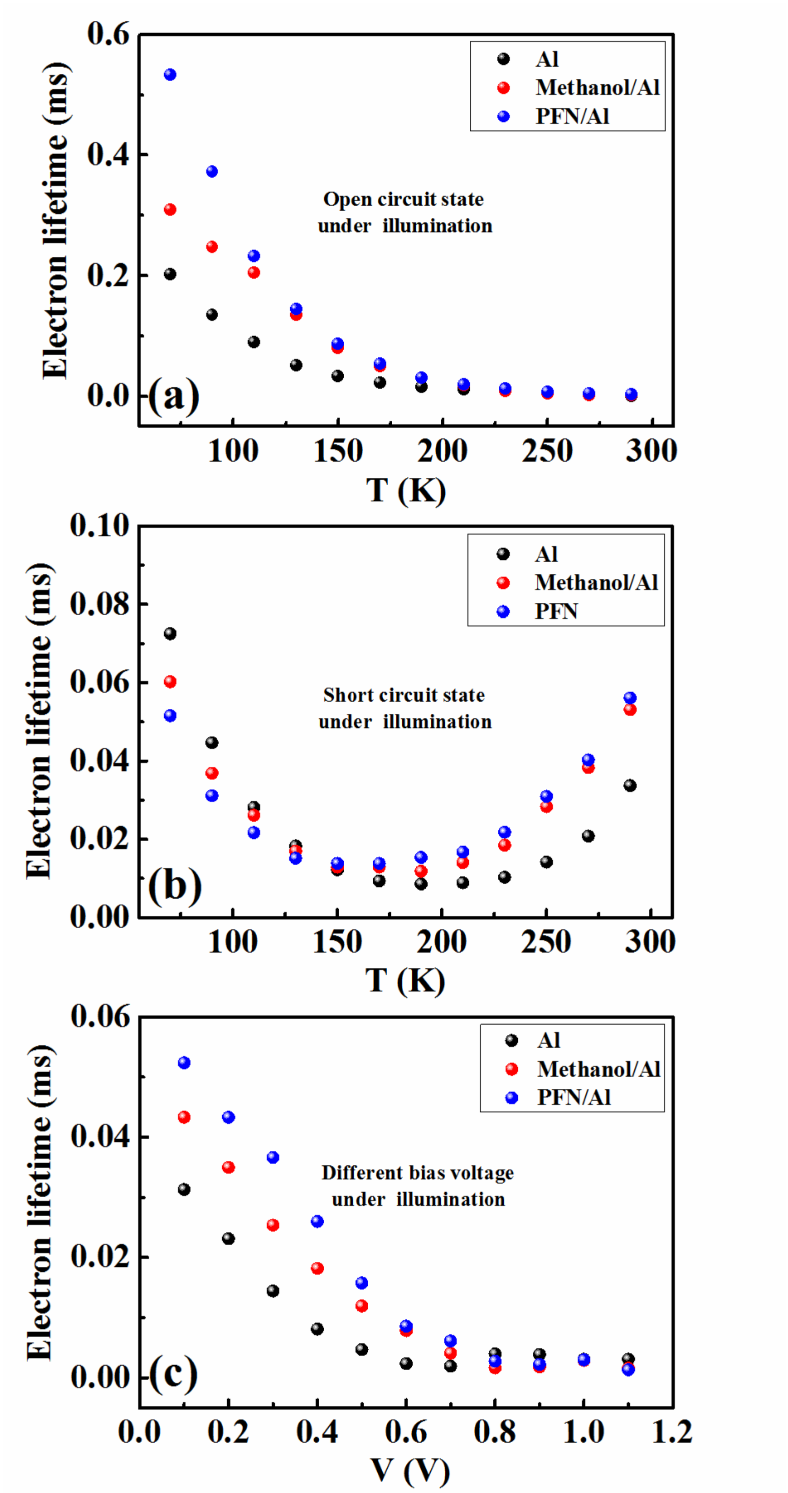

Figure S7 Electron lifetimes of the three kinds of devices under illumination: (a) under open circuit state at various temperatures. (b) under short circuit state at various temperatures. and (c) under various bias voltages at 290 K.

Table S7 Electron lifetimes for the three kinds of devices at various temperatures.

Cathode	Al			Methanol/Al			PFN/Al		
T	τ_n (s)								
	Dark	Open	Short	Dark	Open	Short	Dark	Open	Short
290 K	1.42 $\times10^{-2}$	8.83 $\times10^{-7}$	3.37 $\times10^{-5}$	1.61 $\times10^{-2}$	9.32 $\times10^{-7}$	5.31 $\times10^{-5}$	2.08 $\times10^{-2}$	3.33 $\times10^{-6}$	5.61 $\times10^{-5}$
270 K	1.52 $\times10^{-2}$	4.89 $\times10^{-6}$	2.08 $\times10^{-5}$	1.69 $\times10^{-2}$	2.05 $\times10^{-6}$	3.83 $\times10^{-5}$	2.19 $\times10^{-2}$	5.02 $\times10^{-6}$	4.03 $\times10^{-5}$
250 K	1.83 $\times10^{-2}$	6.75 $\times10^{-6}$	1.42 $\times10^{-5}$	2.21 $\times10^{-2}$	4.87 $\times10^{-6}$	2.84 $\times10^{-5}$	2.63 $\times10^{-2}$	7.44 $\times10^{-6}$	3.10 $\times10^{-5}$
230 K	2.08 $\times10^{-2}$	8.13 $\times10^{-6}$	1.03 $\times10^{-5}$	2.52 $\times10^{-2}$	9.16 $\times10^{-6}$	1.85 $\times10^{-5}$	3.05 $\times10^{-2}$	1.21 $\times10^{-5}$	2.18 $\times10^{-5}$
210 K	2.69 $\times10^{-2}$	1.12 $\times10^{-5}$	8.89 $\times10^{-6}$	3.28 $\times10^{-2}$	1.81 $\times10^{-5}$	1.41 $\times10^{-5}$	3.97 $\times10^{-2}$	1.91 $\times10^{-5}$	1.68 $\times10^{-5}$
190 K	3.47 $\times10^{-2}$	1.56 $\times10^{-5}$	8.60 $\times10^{-6}$	3.97 $\times10^{-2}$	3.01 $\times10^{-5}$	1.18 $\times10^{-5}$	4.88 $\times10^{-2}$	3.08 $\times10^{-5}$	1.54 $\times10^{-5}$
170 K	4.88 $\times10^{-2}$	2.27 $\times10^{-5}$	9.36 $\times10^{-6}$	5.22 $\times10^{-2}$	4.95 $\times10^{-5}$	1.29 $\times10^{-5}$	6.86 $\times10^{-2}$	5.35 $\times10^{-5}$	1.39 $\times10^{-5}$
150 K		3.29 $\times10^{-5}$	1.22 $\times10^{-5}$		7.96 $\times10^{-5}$	1.29 $\times10^{-5}$		8.69 $\times10^{-5}$	1.39 $\times10^{-5}$
130 K		5.12 $\times10^{-5}$	1.83 $\times10^{-5}$		1.35 $\times10^{-4}$	1.70 $\times10^{-5}$		1.44 $\times10^{-4}$	1.52 $\times10^{-5}$
110 K		8.94 $\times10^{-5}$	2.82 $\times10^{-5}$		2.04 $\times10^{-4}$	2.62 $\times10^{-5}$		2.32 $\times10^{-4}$	2.17 $\times10^{-5}$
90 K		1.35 $\times10^{-5}$	4.47 $\times10^{-5}$		2.47 $\times10^{-4}$	3.69 $\times10^{-5}$		3.73 $\times10^{-4}$	3.12 $\times10^{-5}$
70 K		2.02 $\times10^{-4}$	7.25 $\times10^{-5}$		3.10 $\times10^{-4}$	6.02 $\times10^{-5}$		5.32 $\times10^{-4}$	5.16 $\times10^{-5}$

Table S8 Electron lifetimes for the three kinds of devices under various bias voltages at 290 K.

Cathode	Al	Methanol/Al	PFN/Al
Bias voltage	τ_n (s)		
0.1 V	3.13×10^{-5}	4.33×10^{-5}	5.24×10^{-5}
0.2 V	2.31×10^{-5}	3.50×10^{-5}	4.33×10^{-5}
0.3 V	1.44×10^{-5}	2.54×10^{-5}	3.66×10^{-5}
0.4 V	8.10×10^{-6}	1.82×10^{-5}	2.60×10^{-5}
0.5 V	4.69×10^{-6}	1.19×10^{-5}	1.58×10^{-5}
0.6 V	2.37×10^{-6}	7.85×10^{-6}	8.59×10^{-6}
0.7 V	1.93×10^{-6}	4.10×10^{-6}	6.13×10^{-6}
0.8 V	3.99×10^{-6}	1.64×10^{-6}	2.77×10^{-6}
0.9 V	3.88×10^{-6}	1.80×10^{-6}	2.24×10^{-6}
1.0 V	3.09×10^{-6}	2.87×10^{-6}	2.99×10^{-6}
1.1 V	3.09×10^{-6}	1.61×10^{-6}	1.32×10^{-6}

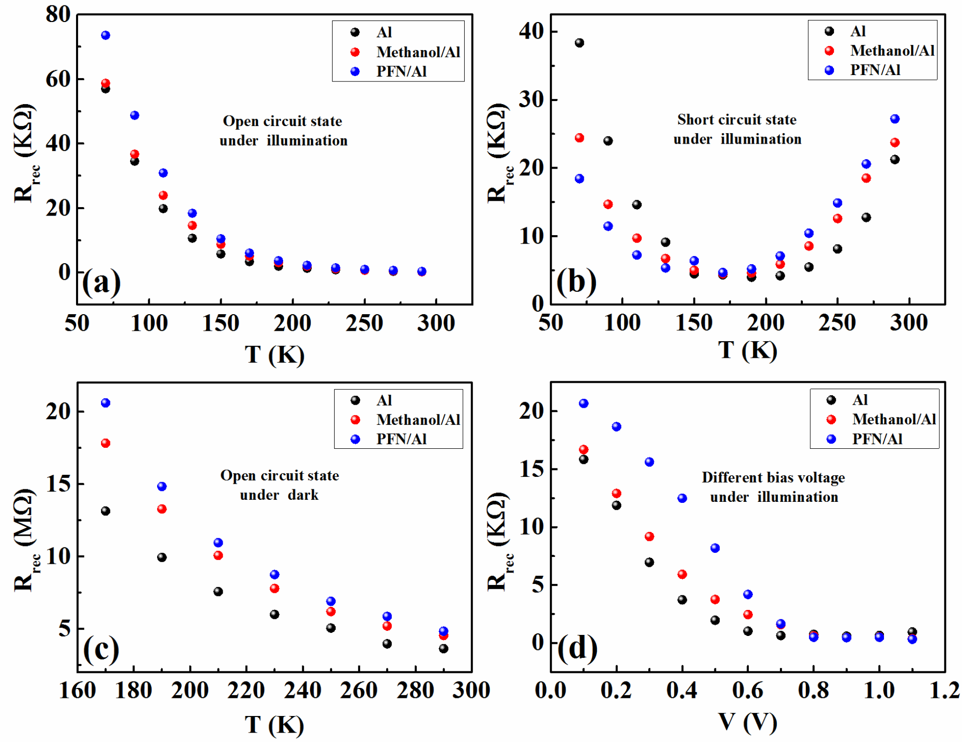

Figure S8 Recombination resistances at various temperatures for the three kinds of devices: (a) at open circuit state under illumination. (b) at short circuit state under illumination. (c) at open circuit state in dark. And (d) recombination resistances at various bias voltages under illumination at 290 K.

Table S9 Recombination resistances for the three kinds of devices at various temperatures.

Cathode	Al			Methanol/Al			PFN/Al		
T	R_{rec} (Ω)								
	Dark	Open	Short	Dark	Open	Short	Dark	Open	Short
290 K	3.62×10^6	219	2.12×10^4	4.53×10^6	240	2.37×10^4	4.83×10^6	371	2.72×10^4
270 K	3.95×10^6	375	1.27×10^4	5.19×10^6	498	1.85×10^4	5.85×10^6	685	2.06×10^4
250 K	5.04×10^6	652	8.12×10^3	6.18×10^6	661	1.26×10^4	6.89×10^6	985	1.49×10^4
230 K	5.99×10^6	893	5.47×10^3	7.79×10^6	1.08×10^3	8.55×10^3	8.74×10^6	1.5×10^3	1.04×10^4
210 K	7.56×10^6	1.28×10^3	4.19×10^3	1.01×10^7	1.85×10^3	5.89×10^3	1.09×10^7	2.26×10^3	7.09×10^3
190 K	9.93×10^6	1.98×10^3	3.98×10^3	1.33×10^7	2.95×10^3	4.60×10^3	1.48×10^7	3.62×10^3	5.18×10^3
170 K	1.31×10^7	3.30×10^3	4.33×10^3	1.78×10^7	5.10×10^3	4.49×10^3	2.06×10^7	6.10×10^3	4.67×10^3
150 K		5.78×10^3	4.49×10^3		8.69×10^3	4.99×10^3		1.05×10^4	6.38×10^3
130 K		1.06×10^4	9.10×10^3		1.45×10^4	6.72×10^3		1.84×10^4	5.34×10^3
110 K		1.98×10^4	1.46×10^4		2.39×10^4	9.70×10^3		3.08×10^4	7.24×10^3
90 K		3.45×10^4	2.40×10^4		3.66×10^4	1.47×10^4		4.87×10^4	1.15×10^4
70 K		5.70×10^4	3.83×10^4		5.87×10^4	2.44×10^4		7.35×10^4	1.84×10^4

Table S10 Electron lifetimes for the three kinds of devices under various bias voltages at 290 K.

Cathode	Al	Methanol/Al	PFN/Al
Bias voltage	R_{rec} (Ω)		
0.1 V	1.58×10^4	1.67×10^4	2.07×10^4
0.2 V	1.19×10^4	1.29×10^4	1.87×10^4
0.3 V	6.95×10^3	9.18×10^3	1.56×10^4
0.4 V	3.72×10^3	5.91×10^3	1.25×10^4
0.5 V	1.96×10^3	3.75×10^3	8.19×10^3
0.6 V	1.02×10^3	2.45×10^3	4.19×10^3
0.7 V	636	1.59×10^3	1.65×10^3
0.8 V	749	613	492
0.9 V	589	464	456
1.0 V	647	513	492
1.1 V	949	370	320
